# IMPRESS: Improved methylation profiling using restriction enzymes and smMIP sequencing, combined with a new biomarker panel, creating a multi-cancer detection assay

**DOI:** 10.1038/s41416-024-02809-1

**Published:** 2024-08-24

**Authors:** Janah Vandenhoeck, Isabelle Neefs, Thomas Vanpoucke, Joe Ibrahim, Arvid Suls, Dieter Peeters, Anne Schepers, Alexander Hoischen, Erik Fransen, Marc Peeters, Guy Van Camp, Ken Op de Beeck

**Affiliations:** 1 Centre of Medical Genetics, University of Antwerp and Antwerp University Hospital, Edegem, Belgium; 2 Centre for Oncological Research Antwerp (CORE), University of Antwerp and Antwerp University Hospital, Wilrijk, Belgium; 3 Department of Pathology, Antwerp University Hospital and University of Antwerp, Edegem, Belgium; 4 Department of Human Genetics and Radboud Institute for Molecular Life Sciences, Radboud University Medical Centre, Nijmegen, the Netherlands; 5 Department of Internal Medicine and Radboud Centre for Infectious Diseases (RCI), Radboud University Medical Centre, Nijmegen, the Netherlands

**Keywords:** Diagnostic markers, Methylation analysis, Cancer epigenetics, Next-generation sequencing

## Abstract

**Background:**

Despite the worldwide progress in cancer diagnostics, more sensitive diagnostic biomarkers are needed. The methylome has been extensively investigated in the last decades, but a low-cost, bisulfite-free detection method for multiplex analysis is still lacking.

**Methods:**

We developed a methylation detection technique called IMPRESS, which combines methylation-sensitive restriction enzymes and single-molecule Molecular Inversion Probes. We used this technique for the development of a multi-cancer detection assay for eight of the most lethal cancer types worldwide. We selected 1791 CpG sites that can distinguish tumor from normal tissue based on DNA methylation. These sites were analysed with IMPRESS in 35 blood, 111 tumor and 114 normal samples. Finally, a classifier model was built.

**Results:**

We present the successful development of IMPRESS and validated it with ddPCR. The final classifier model discriminating tumor from normal samples was built with 358 CpG target sites and reached a sensitivity of 0.95 and a specificity of 0.91. Moreover, we provide data that highlight IMPRESS’s potential for liquid biopsies.

**Conclusions:**

We successfully created an innovative DNA methylation detection technique. By combining this method with a new multi-cancer biomarker panel, we developed a sensitive and specific multi-cancer assay, with potential use in liquid biopsies.

## Introduction

Cancer remains one of the most lethal diseases worldwide. Breast, lung, colorectal, and prostate cancer are amongst the most common cancers, with each over 1.4 million cases per year [1]. Diagnosis typically occurs in an advanced disease stage due to the lack of clear symptoms and the absence of effective screening programs for most cancer types [2]. This is reflected in the percentages of late-stage diagnoses, for example, 68% and 59% in lung and colorectal cancer respectively [3]. Clearly, there is an unfulfilled need for effective diagnostic biomarkers.

An interesting biomarker candidate for cancer detection is DNA methylation. Genome wide epigenetic reprogramming of tumors occurs early in carcinogenesis. Methylation patterns of many tumor types are widely dysregulated compared to those of healthy cells, but the tumor type specific patterns are very distinctive [4–6]. Many studies have investigated the potential of the methylome in recent years but, to date, there are only a few successful methylation biomarkers for cancer in a clinical setting. Our research group has already shown the promise of methylation as a diagnostic biomarker [6]. We demonstrated the capability to discriminate 14 different cancer types from normal tissue and from each other using methylation biomarkers in silico, with high sensitivity and specificity.

It is important to detect biomarkers in a sensitive and specific manner. Currently, bisulfite sequencing is still considered the gold standard for DNA methylation analysis. However, bisulfite is a harsh chemical that degrades DNA, limiting the sensitivity of downstream applications [7]. Alternative bisulfite-free techniques, such as affinity-based methods (e.g. MeDIP-seq, Methyl Cap), are shown to have a lower accuracy compared to bisulfite sequencing [8]. Other bisulfite-free methods include restriction enzymes, which can either be methylation-dependent, digesting only methylated CpG sites (e.g., MspJI), or methylation-sensitive, digesting only unmethylated CpG sites (e.g., HpaII). Recently, two enzymatic technologies for DNA methylation detection were launched, called TET-assisted pyridine borane sequencing (TAPS) and Enzymatic Methyl sequencing (EM-seq) [9, 10]. In TAPS, ten-eleven translocation (TET) oxidation is combined with pyridine borane reduction [10]. EM-seq consists of two conversion steps as well, using TET2 and APOBEC3A [9, 11]. The enzymatic treatment in these techniques is a first step towards eliminating the need for bisulfite conversion [9, 11, 12]. However, genome-wide methylation detection techniques come at a high cost which hampers their implementation in routine diagnostics [13]. Clearly, there is an urgent need for a low-cost, bisulfite-free detection method for the simultaneous analysis of multiple methylation regions that allows accurate prediction of disease.

In this paper, we describe a novel high-multiplex methylation detection technique called IMPRESS (Improved Methylation Profiling using Restriction Enzymes and smMIP sequencing). Methylation-sensitive restriction enzymes (MSREs) have already been used for a very long time for the analysis of methylation in specific regions of the genome [14]. Single-molecule Molecular Inversion Probes (smMIPs) are very efficient for capturing and enriching carefully chosen informative regions of the genome. They are extremely suitable for multiplex analysis of thousands of genomic regions [15]. smMIPs have been used for mutation detection and CNV analyses in different research fields [16, 17] but have never been described for DNA methylation detection. We used this technique for the development of a diagnostic biomarker assay discriminating tumor samples from normal samples. For the selection of the biomarker targets, methylation data of tissue samples from eight of the most lethal cancer types worldwide and normal adjacent tissue were used. Furthermore, methylation patterns of normal blood samples were included for the target selection, ensuring the development of a biomarker panel that is also suitable for use in plasma derived liquid biopsies in the future. In this study, the proof-of-principle of the biomarker assay combining the new IMPRESS technique and the biomarker panel, is described.

## Materials and methods

### Development of new DNA methylation detection technique

**Fig. 1:**
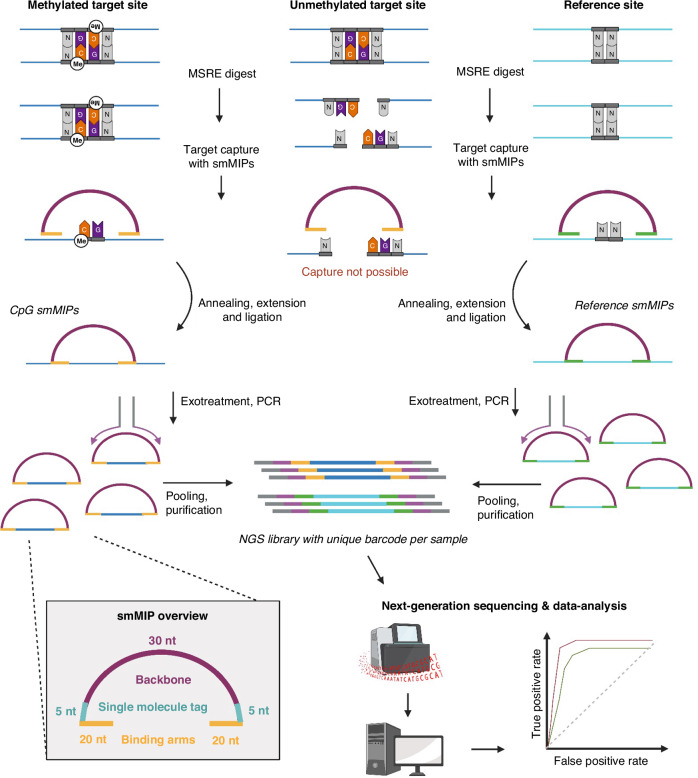
Overview of the IMPRESS technology. During combined digestion with four MSREs, unmethylated recognition sites are digested, whereas methylated CpG sites, blocking the MSREs, remain intact. Subsequently, methylated CpG sites are captured by hybridization of the smMIP binding arms. Both the selected CpG sites and reference sites are targeted by specifically designed smMIPs, referred to as CpG smMIPs and reference smMIPs, respectively. The insert gap is extended and ligated to form a circular fragment. All linear fragments are degraded by an exonuclease treatment and the circular fragments are amplified by PCR with universal primers binding the common smMIP backbone. Eventually, PCR products are pooled, purified and sequenced simultaneously. Created with BioRender.com.

The IMPRESS technique is a novel combination of MSRE digestion and smMIP sequencing. An overview of the technique is given in Fig. 1 and the molecular details are shown in Supplementary Fig. 1. Details of the protocol are described in the Supplementary Methods.

#### IMPRESS technique protocol

In brief, the first step was a combined digestion of 50 ng DNA with four MSREs. The MSREs cleave unmethylated DNA at their recognition sites, while methylated CpG sites block the restriction enzymes, which results in unaffected CpG regions. The methylated CpG regions were captured by the smMIPs through hybridization of the smMIP binding arms. Elongation and ligation of the smMIP created a circular DNA fragment. All remaining linear fragments were degraded by an exonuclease reaction. Thereafter, the circular fragments were amplified by PCR. Finally, all samples were pooled, purified, and sequenced by Next Generation Sequencing. In each sample, lambda phage DNA was spiked in as an internal digestion control.

#### Data analysis pipeline

For the analysis of the NGS output, a bioinformatic pipeline was built using *Snakemake* [18]. Configuration was done in a *json* file, where all pipeline parts can be configured separately. Both MiSeq and NextSeq output can be handled, and as the pipeline has a modular structure, parts can be added or removed very easily. Computational parallelization was achieved per sample for both MiSeq and NextSeq data.

First, the reads were split per sample by using the two sample barcode reads (Supplementary Fig. 1), allowing for one base error per barcode. Reads were then quality trimmed and subsequently mapped to either the human (hg19) or lambda phage genome using the *bwamem* algorithm with default parameters [19]. Next, *Picard MarkDuplicates* was used for duplicate removal, based on the single molecule tags of ten nucleotides in total [20]. Then, reads were filtered out based on mapping quality and mapping flags, keeping only properly paired reads with quality above 15. Finally, reads were counted per smMIP location, only retaining a pair when both reads match the target location within an error margin of 5 base pairs. This way, a dataset with counts for all smMIPs for each sample was obtained.

#### Internal control for digestion

To check whether each sample was sufficiently digested, we used the read counts of the smMIPs targeting spiked-in lambda phage DNA. Two types of lambda phage DNA smMIPs were included: (a) smMIPs targeting a CpG with one of the MSRE recognition sites, and (b) smMIPs targeting a reference site, without recognition site or without CpG site. The percentage of non-digested DNA of each sample was calculated as described below. A threshold of 5% non-digested fragments was set.

$$\begin{array}{c}{{Percentage}\,{of}{non}-{digestion}}_{{digested}\,{sample}}=\\ \frac{\left(\frac{{\Sigma {CpG\; smMIP}\,{count}}_{{digested}\,{sample}}}{\Sigma {{Reference\; smMIP}\,{count}}_{{digested}\,{sample}}}\right)}{{{Average}}_{{all\; undigested\; samples}}\left(\frac{{\Sigma {CpG\; smMIP}\,{count}}_{{undigested}\,{sample}}}{{\Sigma {Reference}\,{smMIP}\,{count}}_{{undigested}\,{sample}}}\right)}\end{array}$$

### Development of multi-cancer biomarker panel

#### Target sites selection

For the development of the multi-cancer detection assay, a panel with candidate methylation biomarkers was built using online available 450 K methylation array data (Table 1). Methylation data processing and analysis were performed based on the methods previously described by Ibrahim et al. [6].

**Table 1:** Amount of samples used for target selection and sequencing experiments.

		Repeatability		Selection of CpG sites*		Multi-cancer assay°		ddPCR validation°	
(Tumor) tissue type	Mortality rank	# Tumor	# Normal	# Tumor	# Normal	# Tumor	# Normal	# Tumor	# Normal
Lung cancer (LUAD + LUSC)	1	3	2	370 + 473	42 + 32	9 + 13	11 + 11	20	22
Colorectal adenocarcinoma (CRC)	2	3	2	411	45	7	10	7	10
Liver hepatocellular carcinoma (LIHC)	4	2	2	377	50	13	11	12	10
Breast invasive carcinoma (BRCA)	5	2	0	791	96	10	9	10	7
Pancreatic adenocarcinoma (PAAD)	6	2	2	184	10	21	24	17	23
Head & neck squamous cell carcinoma (HNSC)	7	2	1	528	50	13	8	13	8
Esophageal carcinoma (ESCA)	8	2	1	185	16	10	5	10	5
Prostate adenocarcinoma (PRAD)	9	2	1	502	50	15	25	14	24
Blood	-	-	-	-	140	-	35	-	22
**Total**		**18**	**11**	**3821**	**531**	**111**	**149**	**103**	**109**

Stomach cancer ranks 3^rd^ in mortality but was left out of the analysis due to a lack of normal samples in the public dataset at the time of analysis.

*LUAD* Lung adenocarcinoma, *LUSC* Lung squamous cell carcinoma.

*CpG sites were selected from online available 450 K methylation array datasets. Tumor and normal tissue sample data were retrieved from The Cancer Genome Atlas (TCGA). Blood datasets, retrieved from the Gene Expression Omnibus (GEO), originate from 18 peripheral blood mononuclear cells (PBMC) (GSE111942), 4 left atrium (GSE62727), 30 erythrocyte progenitors from bone marrow (GSE63409), 6 whole blood, 6 PBMCs, 6 granulocytes, 6 CD4 + T cells, 6 CD8 + T cells, 6 CD56 + NK cells, 6 CD19 + B cells, 6 CD14+ monocytes, 6 neutrophils and 6 eosinophils (GSE35069) and 28 peripheral blood (GSE113012).

°For the multi-cancer assay, we used both matched and unmatched tissue samples. Matched samples are tumor and adjacant normal tissue samples from the same patient. We used 8 matched samples for LUAD, 10 for LUSC, 6 for CRC, 7 for LIHC, 4 for BRCA, 7 for PAAD, 5 for HNSC, 2 for ESCA, and 10 for PRAD. Fot the ddPCR assay, we used most of these samples again.

#### smMIP design

Using the *MIPGEN* software [21], smMIPs were designed for both DNA strands (i.e. double-tiled) for each selected target site. smMIPs in our design contain (a) a common smMIP backbone of 30nt, (b) single molecule tags of 5nt on each side, and (c) two binding arms of circa 20nt that were specifically designed for each target to have an insert size of 50nt (Fig. 1). The single molecule tags differ per smMIP copy and allow filtering for PCR duplicates. Next, smMIPs covering SNPs and/or repeats were removed, and a final selection was made.

### Sample collection and processing

#### Control samples

Lambda phage DNA was purchased from Thermo Fisher Scientific (USA). Human methylated and non-methylated (WGA) DNA was purchased from Zymo Research (USA). Four cancer cell lines were provided by the Centre for Oncological Research (CORE, Antwerp), and one line was purchased from the German Collection of Microorganism and Cell cultures (DSMZ, Germany) (Supplementary Table 1). All cell lines were cultured according to standard protocols from the American Type Culture Collection (ATCC). The cell lines were authenticated at the start of the study and routinely tested for mycoplasma contamination, which was negative. Genomic DNA was extracted using the Blood & Cell culture DNA Maxi kit (Qiagen, Germany). DNA was stored at −20 °C until further use. For the liquid biopsy experiments, cfDNA material was provided by the diagnostics department of the Center of Medical Genetics. This cfDNA was anonymized residual material obtained from NIPT plasma samples. cfDNA was stored at −20 °C until further use. For limited research use of this type of residual material, no additional ethical approval is required.

#### Blood samples

A total of 35 whole blood samples were collected from healthy volunteers. Genomic DNA (gDNA) was extracted using a standard salting-out process. The DNA was stored at 4 °C until further use.

#### Fresh frozen tissue

Tumor tissue and normal adjacent tissue samples were routinely collected by the biobank of the Antwerp University Hospital (UZA, Belgium). All normal tissue cited throughout this manuscript is normal tissue adjacent to tumor tissue, except for the normal breast samples, which originate from breast reductions of healthy women. A total of 225 fresh frozen tissues stored at −80 °C were used (Table 1). Tissue type, presence of invasive tumor, and overall tumor cell percentage (TcP) were verified by a pathologist (D.P.) through microscopic examination of hematoxylin-eosin-stained sections. Samples with a minimum of 5% TcP were retained for analysis. DNA was extracted using the QIAamp DNA Micro Kit (Qiagen, Hilden, Germany) according to the manufacturer’s protocol. The DNA was stored at −20 °C until further use.

### Classifier model construction based on NGS data

For each smMIP, linear discriminant analysis was carried out using the *lda* function from the *MASS* package in the software package R (version 4.0.2) [22]. A model was first constructed and then validated using the *ROCR* package [23]. Five-fold cross validation was carried out with a randomization restriction to proportionally represent the tumor types across the five folds. Predictive accuracy of the LDA models was expressed using the Area under the ROC curve (AUC).

Finally, the least efficient smMIPs were removed with a cutoff of 1000 cumulative counts in all undigested samples, since a minimal number of counts is needed to make a robust classifier. All smMIP models with a cross-validated AUC below 0.8 were removed for the final model. In case of double-tiled smMIPs, the best performing smMIP was selected. All remaining single smMIP models were combined into the final model. The prediction cutoff for each single smMIP model was determined by the lowest sum of false positives and false negatives. The combination of all single predictions was then assessed by a ROC curve and the prediction cutoff was determined based on the highest overall accuracy to produce the final classifier model.

### Validation of the IMPRESS technique with digital droplet PCR

A duplex and triplex ddPCR assay including one and two target sites respectively, as well as a reference site, were developed. These targets overlap with three smMIP targets of our classifier model. For a more detailed description of the assay development and calculations, see Supplementary Methods. The ddPCR assays were used to assess 103 fresh frozen tumor samples and 109 fresh frozen normal adjacent tissue samples (Table 1). These samples were the same as in the IMPRESS experiment, except for the samples with insufficient concentration. For each target site, sensitivity and specificity were calculated based on the methylation level of each sample.

### Statistics and calculations

For the power calculations, we used online datasets to obtain the mean and the standard deviation of the methylation level of the selected targets for the tumor and the normal group. We used the target with the smallest Cohen’s D effect size. A sample size with 67 cases and an equal number of controls holds 80% power to detect any difference between the tumor group (methylation level = 0.50 ± 0.24) and the normal group (methylation level = 0.30 ± 0.20), corrected for multiple testing (1791 CpG sites) with a two-sided test. We were able to collect 111 tumor samples and 149 normal samples. This holds a power of 99%.

For the statistical analyses, differences in average methylation levels between tumor and normal adjacent samples within one tissue type were tested using the Mann-Whitney U test (two-sided). The performance of the IMPRESS and ddPCR was expressed in terms of specificity and sensitivity. Differences in sensitivity and specificity were tested for significance using a test for differences in proportions. To measure the repeatability of our technique, the Pearson correlation between normalized counts from two separate sequencing runs calculated. In addition, a Bland-Altman analysis was performed using the normalized counts of two independent runs.

For all analyses, *p*-values lower than 0.05 were considered significant. All statistical tests were performed in R (version 4.0.2) or GraphPad Prism (version 10.0.0) for macOS, GraphPad Software, Boston, Massachusetts USA, www.graphpad.com.

Normalized counts for each smMIP were calculated as follows:

$$Normalized\,count\,smMIP\,i\,in\,sample\,A=\frac{Absolute\,count\,i\,in\,A}{\sum reference\,smMIP\,counts\,in\,A}$$

Sensitivity, specificity, accuracy and balanced accuracy are calculated as follows with the following abbreviations: true positives (TP), true negatives (TN), false positives (FP) and false negatives (FN).

$${Sensitivity}=\frac{{TP}}{{TP}+{FN}}$$

$${Specificity}=\frac{{TN}}{{TN}+{FP}}$$

$${Accuracy}=\frac{{TP}+{TN}}{{TP}+{TN}+{FP}+{FN}}$$

$${Balanced\; accuracy}=\frac{{Sensitivity}+{Specificity}}{2}$$

### Ethical approval

This study was conducted in accordance with Good Clinical Practice guidelines and the Declaration of Helsinki. Fresh frozen tissue samples used in this study were previously collected in the Biobank of the Antwerp University Hospital and retrospectively used in this study. According to Article 20 of the Belgian Law on the procurement and use of human corporal material intended for human application or scientific research of 19 December 2008, patients provide consent for the use of their bodily material in research when consenting to an invasive procedure. As such, no additional consent was needed for the use of these retrospective samples. For prospectively collected blood samples, informed consent was given by each subject. The study protocol and any modifications thereof were approved by the UZA ethical committee (Ref. N°20/02/056 and Ref. N°41/14/426) before experimental analyses were performed.

## Results

### Development of novel DNA methylation detection technique

#### IMPRESS technique

The first part of this study was the development and optimization of the IMPRESS technique, which combines MSREs and smMIPs [15, 24, 25]. This unique combination allows the use of smMIPs for DNA methylation detection, which has never been described before. An overview of the technique is given in Fig. 1.

The first step was a combined digestion of the DNA with four MSREs (HpaII, HpyCH4IV, AciI and HinP1I). Each MSRE recognition site has a CpG site in the middle (C^CGG, A^CGT, C^CGC and G^CGC). The efficiency of the four MSREs is optimal in the same buffer and at the same temperature, and together they cover 39% of all CpG sites in the human genome [14]. During digestion, unmethylated recognition sites were cleaved. Methylated CpG sites blocked the restriction enzymes which resulted in uncut CpG sites. As a control, undigested samples were also included and treated similarly, except for the omission of MSREs. As a control for digestion, lambda phage DNA was spiked into each sample. After the combined digest, all recognition sites in the lambda DNA were expected to be cleaved, as lambda DNA is not methylated.

In the next step, CpG sites of interest were targeted by a pool of phosphorylated smMIPs. A smMIP is a DNA fragment containing a common backbone of 30nt, single molecule tags of 5nt on each side, and two binding arms of circa 20nt (Fig. 1). Unmethylated CpG regions were cleaved by the MSREs and therefore smMIP capturing was not possible in these regions. Methylated CpG regions remained intact and were captured by the hybridization of the binding arms of the smMIPs. In addition, regions without enzyme recognition sites were targeted as a reference. Elongation and ligation of the smMIP created a circular DNA fragment. In all capture reactions, some smMIPs were ligated without a 50nt insert. These so-called empty smMIPs were eventually removed through purification and through filtering during data analysis.

After the capture reaction, an exonuclease treatment was performed, in which all linear fragments such as unbound smMIPs or original DNA strands were degraded, and only circular fragments remained intact. These fragments were amplified by PCR. Finally, all fragments were purified and sequenced by NGS.

In addition to our wet lab protocol, an accompanying bioinformatics analysis pipeline was developed to process the NGS data. Using a *Snakemake* workflow, all sequencing reads were deconvoluted per sample and mapped to the genome. Next, all duplicates were removed. After quality filtering, reads per smMIP location were counted for each sample and a counts table was obtained.

#### Efficiency of the methylation-sensitive restriction enzymes

To test the cutting efficiency of the combination of the four selected MSREs, both a methylated and an unmethylated lambda DNA sample were digested by the MSREs. Subsequently, the digested samples were amplified in triplicate with primer pairs hybridizing around one of the MSRE recognition sites in a qPCR experiment. Undigested, (un)methylated lambda DNA samples were also included as positive controls.

**Fig. 2:**
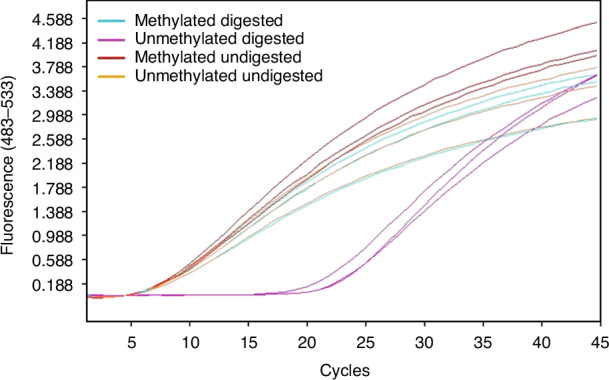
Efficiency of the methylation-sensitive restriction enzymes. Amplification curve of qPCR of (un)methylated and (un)digested lambda DNA. This is an example of one primer pair hybridizing around a MSRE recognition site. Two types of control samples were used, unmethylated lambda DNA and artificially methylated lambda DNA by CpG methyltransferase M.SssI (New England Biolabs). Each sample was amplified in triplicate. Methylated digested (blue), unmethylated digested (purple), methylated undigested (red) and unmethylated undigested (orange) samples have an average Ct value of 5.7, 22.0, 5.0 and 5.0, respectively.

According to the Lightcycler software (Roche), the undigested samples had an average Ct value of 5.0 and the unmethylated digested sample had an average Ct value of 22.0 (ΔCt=17) (Fig. 2). This means that merely 1 in 2^17^ = ~ 131,000 DNA molecules were not digested. Thus, the remaining fraction of undigested DNA in unmethylated samples is negligible. The methylated digested sample had an average Ct value of 5.7, indicating that methylation effectively blocks digestion by the MSREs.

#### Repeatability

**Fig. 3:**
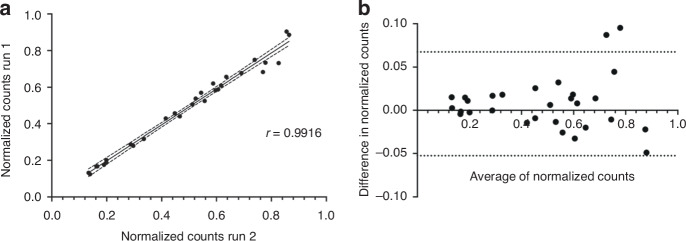
Repeatability of novel IMPRESS technology. Repeatability of the technique by comparing two independent experiments with the same samples. **a** The normalized counts of the samples have a correlation coefficient of *r* = 0.99. **b** Bland-Altman comparison for the repeatability. Bias is 0.007502 ± 0.03066 with 95% limits of agreement [-0.05259; 0.06759]. Plotting was performed using GraphPad Prism.

To evaluate the repeatability of the IMPRESS technique, two independent experiments were performed on a set of 29 fresh frozen tissue samples (Table 1). We used the smMIPs designed for the multi-cancer biomarker panel (see Development of a multi-cancer biomarker panel). The two libraries were sequenced together, and data processing was performed. The normalized counts (calculation in Statistics and calculations) of the same samples for both experiments were compared, and a Pearson correlation coefficient of r = 0.99 was obtained (Fig. 3a). In the Bland-Altman analysis, the bias was 0.007502 ± 0.03066 with 95% limits of agreement of −0.05259 and 0.06759 (Fig. 3b). These results prove the high repeatability of the IMPRESS technique.

### Development of a multi-cancer biomarker panel

**Fig. 4:**
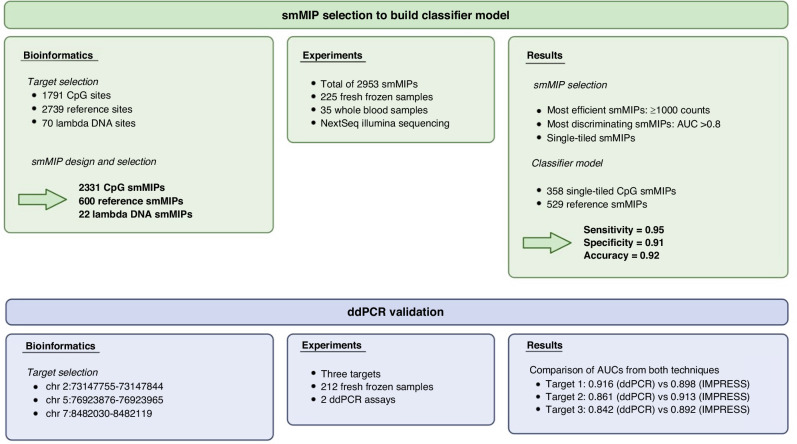
Overview of the main analyses for the multi-cancer detection assay. The two main experiments are shown: One experiment by IMPRESS and one experiment by digital droplet PCR (ddPCR). The first experiment is used for the determination of the final classifier model. The second experiment validates the IMPRESS technology by the gold standard ddPCR technique. Created with BioRender.com.

The second part of this study was the development of a multi-cancer diagnostic biomarker panel, which could subsequently be validated by the IMPRESS technique to develop a multi-cancer detection assay. For the selection of potential biomarkers, online available methylation data of eight of the most lethal cancer types worldwide and healthy blood samples were used (Table 1). In total, 1791 hypermethylated CpG sites (Fig. 4) were selected based on the following parameters: (a) a minimum average methylation level of 0.5 in the tumor tissue samples of eight cancer types, (b) a maximum average methylation level of 0.3 for the normal adjacent tissue and blood, and (c) the presence of at least one restriction site for one of the four used MSREs. On average, four recognition sites were interrogated per region. Normal blood datasets were included for biomarker selection, resulting in a biomarker panel that is suitable for liquid biopsies as well. Secondly, a total of 2739 reference sites were selected from the human genome. These reference sites were included to estimate the effective total amount of input DNA and allow normalization of the results per sample. Reference sites were chosen to (a) not contain a recognition site of the selected MSREs (1000 sites), or (b) not contain a CpG site (1,739 sites). These regions are never cleaved by the enzymes and are therefore always captured by the smMIPs. Lastly, both CpG (10 per MSRE site) and reference sites (15 without recognition site, 15 without CpG site) were selected in lambda phage DNA. Lambda phage DNA is never methylated and is used as an internal control for the enzymatic digestion reaction.

For these 1791 hypermethylated CpG sites, 2739 reference sites, and 70 lambda phage DNA sites, smMIPs were designed for both DNA strands (Fig. 4). After the removal of smMIPs covering SNPs and/or repeats, 2331 CpG smMIPs and 600 reference smMIPs (300 without recognition site and 300 without CpG site) were selected for human targets. For lambda phage DNA, 12 CpG smMIPs (3 per restriction site) and 10 reference smMIPs (5 without recognition site and 5 without CpG site) were selected.

### Multi-cancer detection assay

#### Data exploration

To evaluate the biomarker panel and the IMPRESS technique, we performed an experiment on fresh frozen tissue and blood samples (Fig. 4). First, we prepared a sequencing library with 111 tumor samples, 114 normal adjacent tissue samples and 35 whole blood samples (Table 1) targeted by a total of 2953 smMIPs (2331 CpG smMIPs, 600 reference smMIPs and 22 lambda smMIPs). Capillary electrophoresis analysis of this library is shown in Supplementary Fig. 2. Sequencing was performed on the Illumina NextSeq system and NGS parameters are shown in Supplementary Table 2. After the first analysis of the raw data, read counts for all smMIPs for each sample were obtained. Based on the characterization experiments, a minimum read count threshold of 5000 counts per sample was determined, and all samples met this requirement (Supplementary Table 3).

**Fig. 5:**
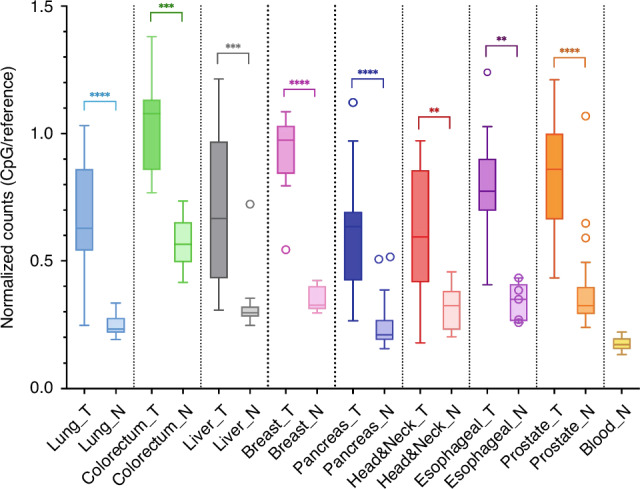
Sample distribution of normalized counts for all target sites. All nine different tissue types are displayed in distinct colors in the Tukey boxplot. For every tissue type, the sample distribution of the sum of the normalized counts for tumor (T) and normal adjacent tissue (N) are shown. This value is the ratio of the sum of the counts of the CpG smMIPs and the sum of the counts of the reference smMIPs. Significance is indicated with asterisks: * = *p*-value ≤ 0.05, ** = *p*-value ≤ 0.01, *** = *p*-value ≤ 0.001 and **** = *p*-value ≤ 0.0001. Mann-Whitney U test was performed using GraphPad Prism.

The efficiency of the MSRE digest was verified in each sample by the spiked-in lambda phage DNA as an internal control (calculations in part 2.1.3). A threshold of 5% non-digested fragments was set. One out of 260 samples exceeded this threshold (9.7%) and was removed from further analyses. On average, only 1.3% of the DNA in each sample was not properly digested (Supplementary Figure 1). In total, 19 underperforming CpG smMIPs with no counts in any sample were removed from the analysis. Finally, normalization was executed per sample to correct for the amount of input DNA. Hereto, we divided the counts of every CpG smMIP by the sum of all reference smMIP counts, resulting in a final dataset with counts of 2,312 CpG targeting smMIPs for 259 samples. The normalized count is assumed to be higher in samples methylated for our targets (i.e., tumor samples) and lower in samples unmethylated for our targets (i.e. normal samples). An overview of the sample distribution of the sum of the normalized CpG smMIP counts is given in Fig. 5. Tumor samples showed higher and more spread normalized counts, while normal samples showed lower and more similar normalized counts. However, the normalized counts for normal colorectal tissue are higher than all other normal tissues and overlap with some of cancer types. The blood samples had the lowest normalized counts compared to all other tissue types. Within each tissue type, the average normalized counts of tumor and normal samples were significantly different.

#### Selection of the most efficient and discriminating smMIPs

**Fig. 6:**
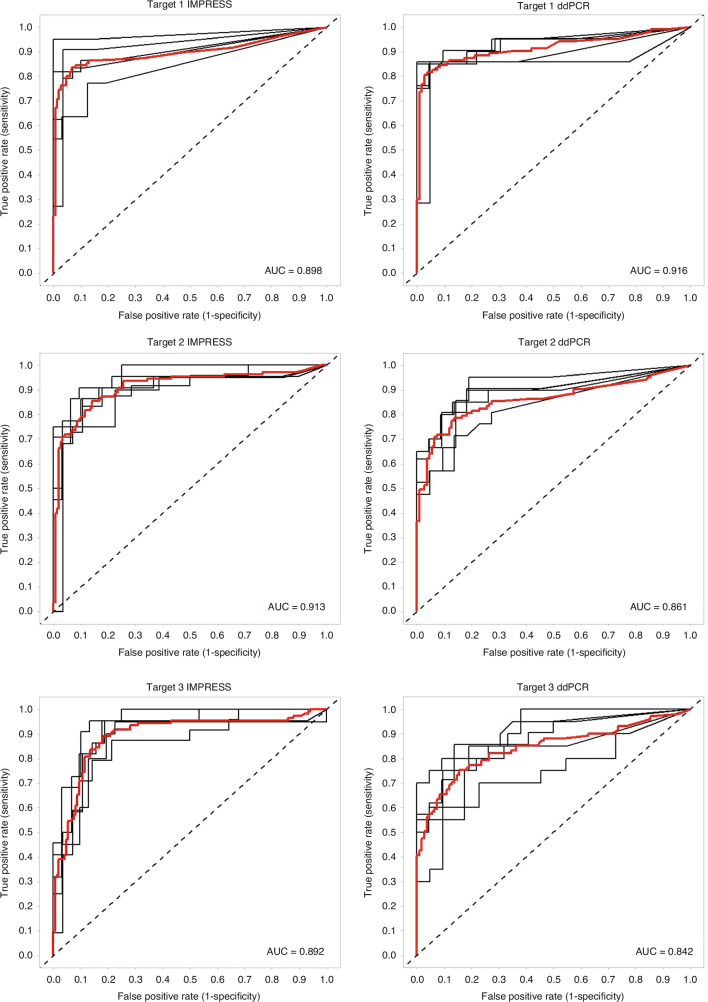
ROC curves of the three selected target sites for both the IMPRESS assay and the ddPCR assay. The left panels are the ROC curves of the IMPRESS assay. The right panels are the ROC curves of the ddPCR assay. For each model, the 5-fold cross validation ROC curves (black) and the mean ROC curve (red) are plotted. Both assays target 3 CpG sites: location chr2:73147755-73147844 (target 1), chr5:76923876-76923965 (target 2) and chr7:8482030-8482119 (target 3). AUC values are shown for each ROC curve.

To determine the final biomarker panel for the classifier model, the most efficient and discriminating smMIPs were selected. Using the final dataset with counts of 2,312 CpG targeting smMIPs for 259 samples, a single smMIP linear discriminant analysis (LDA) model was constructed using 5-fold cross validation and the mean cross validated AUC (cvAUC) was calculated for each smMIP. ROC curves for three selected smMIPs are shown in Fig. 6 (left panels). These smMIPs target the same regions as the ddPCR validation assays (see 3.4.2). The distribution of cvAUC is shown in Supplementary Fig. 4. We used a cvAUC cutoff of 0.8 for selecting the best differentiating smMIPs, and a cutoff of 1,000 cumulative counts per smMIP in all undigested samples as a measure for smMIP efficiency. This resulted in a set of 511 CpG smMIPs. Additionally, for CpG sites targeted by multiple smMIPs (i.e. double-tiled), the best performing one was selected. The 511 remaining CpG smMIPs targeted 358 CpG sites. Of these sites, 153 were targeted double-tiled. The difference in cvAUC between smMIPs targeting the same CpG site was less than 0.05 for 84.3% of the multi-targeted CpG sites. The correlation coefficient of these cvAUC values is r = 0.668 (Supplementary Figure 5). Finally, 358 single-tiled CpG smMIPs remained for further analyses (Supplementary Table 4). For the reference smMIPs, only the efficient smMIPs were selected, with the same count cutoff of 1,000 cumulative counts in all undigested samples. As a result, 529 reference smMIPs were selected.

#### Classifier model

The remaining 358 CpG smMIP models were then combined into a single model by first selecting the cutoff for every single model for which the sum of false positives and false negatives was the lowest. Then all predictions were combined, and the cutoff was selected based on the highest overall accuracy, which was achieved when 114 single smMIP models agreed on a tumor classification. This final model has a sensitivity of 0.95, a specificity of 0.91, and an accuracy of 0.92 (Table 2).

**Table 2:** Metrics of our classifier model.

	Multi-cancer	Lung	Colorectal	Liver	Breast	Pancreas	Head and neck	Esophagus	Prostate	Blood
**True positives**	104	21	7	11	10	19	11	10	15	-
**True negatives**	135	22	1	10	8	22	5	5	24	35
**False positives**	14	0	9	1	1	2	0	0	1	0
**False negatives**	6	1	0	2	0	1	2	0	0	-
**Sensitivity**	0.945	0.955	1.000	0.846	1.000	0.950	0.846	1.000	1.000	-
**Specificity**	0.906	1.000	0.100	0.909	0.889	0.917	1.000	1.000	0.960	1.000
**Accuracy**	0.923	0.977	0.471	0.875	0.947	0.932	0.889	1.000	0.975	-
**Balanced accuracy**	0.926	0.977	0.550	0.878	0.945	0.934	0.923	1.000	0.980	-

The multi-cancer column combines the results of all tumor types.

In addition, we investigated the results per cancer type (Table 2). Accuracy per cancer type ranged from 0.88 to 1, with the exception of colorectal cancer, which performed significantly worse than all other types (accuracy of 0.47). Sensitivity was very high overall, with only 6 false negatives among liver, pancreas and head and neck tumors. False positives are attributed to five tumor types, with colorectal tumors among the highest (specificity 0.10), which skews the overall specificity. However, when exclusively investigating colorectal samples, the cutoff can be adjusted to 282 single smMIP models to obtain a classification accuracy of 1. Interestingly, healthy blood samples never showed up as false positives.

### Validation with digital droplet PCR

We validated the IMPRESS technique with the gold standard ddPCR technology. Therefore, we selected three target sites of our final classifier model (see 3.3.3). For this selection, we started with the top 200 best performing smMIP targets, for which we aimed to design ddPCR primers and probes. Finally, we selected the three targets with the best performing primers and probes for the development and optimization of two ddPCR assays (see Supplementary Methods). The assays were executed on 103 tumor samples and 109 normal adjacent tissue samples that were used for the IMPRESS experiments (Fig. 4). For each of the targets, a single model was built to evaluate the sensitivity and the specificity. In Fig. 6 the ROC curves for the three target sites are shown both for the IMPRESS assay and for the ddPCR assay.

The sensitivity and specificity of the single ddPCR models were compared with those from the single smMIP models for the three targets. Figure 6 and Supplementary Table 4 show that only minimal differences between both technologies are found, admittedly favoring the IMPRESS technique. These results indicate that the IMPRESS technique performs at least equally well as the gold standard ddPCR.

### Potential for liquid biopsies

**Fig. 7:**
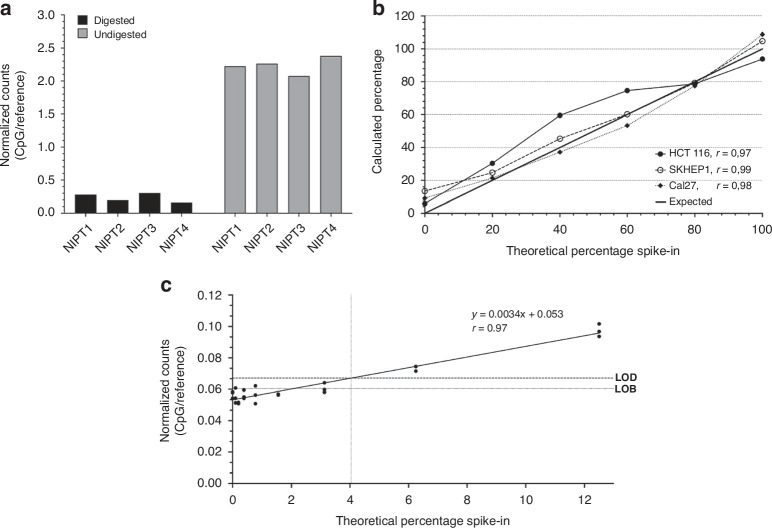
Potential for liquid biopsies. **a** Normalized counts of digested and undigested cfDNA samples. Four cfDNA samples were used to verify that the protocol works in liquid biopsies. On the left side, digested samples are shown. On the right side, undigested samples are shown as positive controls. The DNA input amount is 5 ng. **b** Calculated percentages based on normalized counts for all target sites of normal cfDNA with spiked-in cell line DNA. Sheared cell line DNA was spiked into normal cfDNA at 0-20-40-60-80-100%. The percentages are calculated by the linear regression through the normalized counts for each sample. The expected value ranges from 0% to 100%, although it is known that normal samples are not 0% methylated in all our targets. The correlation of the calculated and expected percentages is given by r for each cell line. See Supplementary methods for calculations. **c** Limit of methylation detection calculated by the normalized counts for all target sites. Fully methylated human DNA was spiked into unmethylated human DNA at 0-0.10-0.20-0.39-0.78-1.56-3.13-6.25-12.5% in triplicate. The linear regression through the normalized counts for each sample has a Pearson correlation coefficient of *r* = 0.97. The limit of blank (LOB) is 0.060 and the limit of detection (LOD) is 0.067. The methylation percentage corresponding to the LOD is 4.04%. Linear regression and plotting was performed using GraphPad Prism.

To test whether the technique holds potential for use as a multiplex tool for methylation detection in liquid biopsy samples, several characterization experiments were performed. The results are described below and are shown in Fig. 7.

#### Determination of the amount of input DNA

To test the possibility of lowering the DNA input amount of the IMPRESS technique, we tested different reaction conditions for the MSRE digestion and the smMIP sequencing. The results indicate the feasibility of lowering input amount to 5 ng and 10 ng DNA for the digest and the smMIP sequencing, respectively (lowest amounts tested) (Supplementary Methods and Supplementary Figure 6).

#### Cell free DNA

To further explore the lower limits of input DNA and the feasibility of the technique to study liquid biopsies, four cell-free DNA (cfDNA) samples were tested with 5 ng of input. Besides MSRE digested samples, undigested samples were also included as positive controls. The samples were captured by smMIPs and sequenced according to our protocol. The sequencing results were analyzed using our in-house developed pipeline and read counts were obtained for the 2331 CpG smMIPs and 600 reference smMIPs. To normalize for the DNA input, the sum of CpG smMIP counts was divided by the sum of reference smMIP counts for each sample. Results showed that digested samples had an average normalized count of 0.24 while the undigested samples had an average normalized count of 2.24 (Fig. 7a). This indicates that the samples were effectively digested by the MSREs as well as efficiently captured by the smMIPs and sequenced.

To mimic the presence of circulating tumor DNA (ctDNA) in cfDNA, DNA from three tumor cell lines was sheared into fragments of 150–500 bp and spiked into cfDNA samples in different percentages between 0% and 100%. The calculated percentages based on the normalized counts (see Supplementary methods) were closely correlated to their expected value, with a correlation coefficient r of 0.97, 0.99 and 0.98 for the three different cell lines (Fig. 7b). This indicates the applicability of the technique for the quantification of methylation. The calculated percentage of 20% spiked-in DNA (lowest percentage tested) ranged from 21% to 30%, while those of 0% spiked-in DNA (only cfDNA) ranged from 6% to 13%. This means samples with 20% spike-in of cell line DNA had on average a threefold higher percentage of normalized counts than cfDNA samples without spike-in.

#### Limit of methylation detection

To determine the limit of methylation detection, we spiked human fully methylated DNA into non-methylated DNA in different percentages between 0% and 12.5% in triplicate. All samples were digested, captured by smMIPs and sequenced following our protocol. The samples with a low methylation level only resulted in background signal. We calculated the limit of blank (LOB) and limit of detection (LOD) following the formulas described by Armbruster et al. [26] to determine the lowest detectable methylation level. The LOD corresponds to a methylation level of 4.04% (Fig. 7c). For samples with methylation levels above 4.04%, the measurements will exceed the background signal.

## Discussion

In this study, we successfully created a novel methylation detection technique by combining MSRE digestion with smMIPs. Although digestion of DNA with MSREs has been described since 1978 [27] and has been standard practice for the past decades, smMIPs were only described less than ten years ago [15]. To date, the use of smMIPs was limited to the detection of DNA mutations, microsatellite instability, gene amplifications and differential expression (cDNA) [16, 24, 28]. In this regard, smMIPs have frequently been proposed for use in routine diagnostics for cancer detection in recent years. In concordance with our results, several studies have demonstrated the high sensitivity of smMIP-sequencing, as well as the possibility to use limited amounts of material [16, 17, 28–31]. smMIPs have also demonstrated their utility in other research fields [32, 33]. smMIP panels can be easily adapted towards their intended application, which is extremely useful with the rapid expansion of molecular markers in all fields [16]. Taken together, this places our novel technique as a promising and widely applicable epigenetic tool for the detection and follow-up of many diseases.

We developed a multi-cancer methylation biomarker panel, and we validated this panel by combining it with the IMPRESS technique, resulting in a robust multi-cancer detection assay. With an overall cross-validated accuracy of 0.92, this final model performed very well in classifying samples. The overview of the sample distribution (Fig. 5) shows a spread of tumor samples for most of the tumor types, while normal tissues are grouped closer together. There was no correlation between tumor cell percentage and normalized counts (data not shown). The spread of tumor samples is most noticeable for head and neck, and liver tumors. The former is a heterogeneous group of locations and cell types, which can account for this spread. For liver tumors, there is only one cell type, but there was a lot of variety in the patient group. Clinical records show that some of the samples originated from patients with alcohol abuse and/or patients with hepatitis. This could potentially have affected the methylation levels in the liver.

Most false positive predictions are the result of normal adjacent colorectal tissue samples having a higher methylation for our targets than the other normal tissue groups (Fig. 5). This could be due to field cancerization, which causes (epi)genetic alterations in histologically normal-looking tissue adjacent to cancerous lesions [34]. Although this phenomenon has also been described in other cancer types, this is not clearly seen in our analyses. In Fig. 5, colorectal samples have the highest methylation rate, both for normal samples and tumor samples. As a result, perfect separation of tumor and normal samples is observed when taking only colorectal samples into account, while many normal colorectal samples are false positives in the overall model. This suggests that colorectal cancer might not be a suitable addition to a multi-cancer assay utilizing this biomarker panel, despite its potential differentiating ability within colorectal samples.

While the final model does not make a correct prediction for all samples (0.95 sensitivity and 0.91 specificity), for every sample there are single-smMIP models that do. This emphasizes the exceptional performance of our biomarker panel and confirms the presence of significant methylation differences across all samples.

This model was specifically constructed to include many CpG sites because we intend to use this assay in liquid biopsies in the future. There, only a limited amount of ctDNA is available in the cfDNA, especially in early tumor stages. This ctDNA is fragmented and chances are small that the whole tumoral genome is covered in a liquid biopsy sample.

With respect to liquid biopsies, we performed some additional characterization experiments to thoroughly test the amount of input DNA for the IMPRESS technique. For the digestion reaction, the performance of the MSREs remained equal when lowering the input amount to 5 ng. We also showed that an input of 10 ng can discriminate between tumor cell lines and healthy blood samples equally well as 20 ng and 100 ng (Supplementary Figure 6). Moreover, the use of 5 ng cfDNA has been successfully tested in our lab (Fig. 7a). This is extremely important in a liquid biopsies context, where often even less than 5 ng is available. To mimic cfDNA, we experimented with sheared tumor cell line DNA that was spiked into normal cfDNA samples. The results demonstrated the possibility to discriminate 20% spiked-in tumor DNA from normal cfDNA. The high correlation coefficients indicated the quantification potential of the technique. However, the counts value is always a relative number and not an exact methylation percentage, as the majority of the targeted regions contain several CpG sites. In theory, we only sequence targets in which all the CpG sites are methylated and as such, we measure the count of all fully methylated fragments. Finally, we did a limit of detection experiment. As there was some background signal, we determined the methylation value for which the measurement exceeded the background signal. This limit of methylation detection was 4.04%. A potential explanation for the background signal is incomplete MSRE digestion. We know that a small percentage of the DNA is not properly digested. This is estimated to be 1.3% by the internal control. In addition, commercially purchased 0% methylated DNA was used, although previous data (not shown) indicate that a small degree of methylation is still present.

In recent years, multi-cancer detection (MCD) has gained more interest. The IMPRESS technique combined with the multi-cancer biomarker panel shows great potential in this field. Most MCD tests are described for liquid biopsies, which is yet to be done for IMPRESS. Nevertheless, preliminary results show that IMPRESS could become an important and novel addition to the liquid biopsy field.

Cohen et al. described their CancerSEEK test in 2018. They detected both circulating proteins and mutations in cfDNA of eight different cancer types [35]. Although mutations and proteins have often been the first choice when developing biomarker assays, it has become clear that methylation signatures possess some major advantages compared to the former. Methylation occurs very early in cancer development, possibly before actual neoplastic transformation, which renders it especially interesting for early diagnosis. Given that no prior knowledge is needed of the tumor molecular profile, methylation biomarkers are more universal than mutation markers. Since methylation-based tests can be used off the shelf, they are much faster and cheaper to use [36]. The utility of methylation signatures was for example demonstrated by Chen et al., who published the PanSeer test in 2020. The results obtained with their blood-based test show that cancer is detected in 95% of asymptomatic patients for five cancer types. Although further investigation is needed to confirm the results, they claim that several cancers can already be detected four years prior to diagnosis, based on methylation biomarkers[37], which is very promising for early cancer detection. Others have worked on the use of methylation signatures for screening and early detection as well. The biotechnology company GRAIL finances several clinical trials where the use of their Galleri^®^ test is evaluated. These trials were started based on publications by Liu et al. and Klein et al., who first tested and independently validated the performance of targeted methylation analysis of cfDNA for multiple cancer types [36, 38]. Both for the PanSeer as well as for the first experiments with the Galleri^®^ test, bisulfite-based technologies were used [36–38]. We believe that the sensitivity in cfDNA could be increased with our novel technique using MSREs, as we avoid bisulfite conversion, which is a harsh chemical treatment of DNA.

The use of enzymes has recently been gaining more attention. A prime example is the recent development of the Enzymatic Methyl sequencing (EM-seq) technology. This EM-seq technology is for example used for targeted methyl-seq in combination with the Twist Human Methylome Panel (Twist Bioscience). In a limited number of publications, EM-seq is described to be superior to WGBS for sensitivity, repeatability and base composition [9, 11, 12]. However, EM-seq requires a large investment for library preparation [9]. TAPS was also recently described in a few papers [10, 39, 40]. However, there are no external publications comparing TAPS to other state of the art technologies in literature. Considering smMIPs, Arts et al. already described the low cost of smMIP-sequencing, which does not drastically increase with MSRE treatment. Therefore, our novel technique is more cost-effective compared to current bisulfite-free alternatives on the market [24].

The application of the IMPRESS technique lies in targeted biomarker sequencing. This type of targeted sequencing technology is becoming more popular, and our technique can become an important new platform to be used in this area. IMPRESS enables the multiplexing of a considerable number of target sites, extending to thousands, in contrast to methodologies like droplet digital PCR (ddPCR), which offer limited multiplexing capabilities. Additionally, compared to genome-wide techniques, our approach presents a distinct advantage in terms of cost-effectiveness. By selectively analyzing predefined sites of interest, we substantially decrease sequencing costs. The technique is easily implementable in standard equipped laboratories. We use widely available reagents, and the protocols are straightforward, making the technique easily applicable for research groups with access to NGS infrastructure. The hands-on time from DNA extraction to sequencing is approximately 4.5 h, which is comparable with EM-seq [41]. Moreover, by sequencing the insert fragment of interest, we can detect and correct for nonspecific hybridization. Notably, our technique boasts high throughput, facilitating the simultaneous analysis of 9x384 samples within a single sequencing run. Depending on the desired coverage per sample, a larger sequencing kit may be required. Furthermore, this technique has the potential for integrating different genetic information into one assay, for example, mutation and CNV analysis. smMIPs could be designed for any target type, taking the MSRE recognition sites into account, and combined into a single assay. As such, one single experiment could provide the information that is now only obtained after several analyses.

There are a few limitations in this study. The most important one is that we have not yet tested liquid biopsies from cancer patients. However, our results show that blood samples register very low normalized counts by the IMPRESS technique for our biomarker panel. In addition, we can efficiently use 5 ng of cfDNA as input, and there is a limit of methylation detection of 4.04%. In the future, more optimization steps will be executed, and liquid biopsy samples from cancer patients will be tested.

Another limitation is that only 39% of the CpG sites of the methylome are located in recognition sites of the enzymes used in our assay [14]. This could easily be solved by using other or additional restriction enzymes, through which a large majority of CpG sites in the genome can be made available for analysis.

Furthermore, we could not include tissue from fully healthy persons, instead, we used normal tissue collected at a distance from the tumor. Clinical records do not show the exact distance. In the literature, it has been described that tissue samples adjacent to a tumor might have molecular alterations (e.g. field cancerization) but look microscopically like normal tissue [42, 43]. This might result in a more difficult discrimination between normal and cancer tissue by our classifier model.

Lastly, we did not yet include cancer type-specific smMIPs for the detection of tissue-of-origin. Classification of cancers of unknown primary is an important aspect of cancer diagnostics and is increasingly described in the literature. It can already be determined based on in silico analyses [44] and therefore, including TOO determination in our assay is an important step for the future. Analyses have been performed in our research group to determine tissue-specific methylation patterns, for which smMIPs will be designed and included in the future. This will not cause any problems as we have already demonstrated the very high multiplex capacity of our technique.

In conclusion, we developed a novel method for sensitive detection of DNA methylation, we developed a multi-cancer methylation biomarker panel, and we combined those two into a multi-cancer detection assay. Our characterization experiments already demonstrate the application of this technique and the biomarker panel for low amounts of fragmented DNA. The combination of multiple markers covered by the smMIPs allows for the high sensitivity that is essential in liquid biopsies and early cancer detection applications.

## Supplementary information

Supplementary information

Suppl Table 3

Suppl Table 4

## Data Availability

The data that support the findings of this study are available on request from the European Genome-Phenome Archive (EGAS00001007559).

## Code availability

Code, including all version information, is available upon request as a code ocean capsule, where the code can be run in an online environment or downloaded (10.24433/CO.5112387.v1).
