# Supplementary material for: IMPRESS: Improved methylation profiling using restriction enzymes and smMIP sequencing, combined with a new biomarker panel, creating a multi-cancer detection assay
Source: Br J Cancer. 2024 Aug 24;131(7):1224–36. doi: 10.1038/s41416-024-02809-1 (PMC11442765; doi:10.1038/s41416-024-02809-1)
Supplement: Supplementary file 1 — Supplementary information [file 41416_2024_2809_MOESM1_ESM.docx]

# **Supplementary information**

**Supplementary methods: IMPRESS protocol**

1. *MSRE digest*

The first step was a combined digestion of the DNA with four MSREs (HpaII, HpyCH4IV, AciI and HinP1I). The MSREs digest unmethylated DNA at their recognition sites (C^CGG, A^CGT, C^CGC and G^CGC, respectively). Methylated CpG sites block the restriction enzymes which results in unaffected CpG regions.

In a total digestion volume of 10µL, an input of 50ng DNA of each sample was diluted in 8.1µL H_2_O (on ice). Per sample, a mastermix was added containing 1µL CutSmart Buffer (New England Biolabs), 0.5µL EcoRI digested lambda DNA (0.75pg/µL) (Thermo Fisher Scientific), and 0.1µL of each restriction enzyme HpaII, HpyCH4IV, AciI and HinP1I (10U/µL) (New England Biolabs). Lambda DNA was digested by FastDigest EcoRI (Thermo Fisher Scientific) according to the manufacturer’s protocol to remove concatemers and spiked-in as an internal control. The reaction was incubated for 16 hours at 37°C, followed by a heat inactivation step for 30 min at 65°C and stored at 4°C until continuation. To enable downstream calculation of the efficiency of our smMIP-pool and the percentage of non-digestion, undigested samples were also included. A modified version of the protocol was executed for all these samples, where the 4 MSREs were replaced by H_2_O. All undigested doubles were incubated as described above.

1. *Pooling and phosphorylation*

One smMIP pool was made by adding the 2953 smMIPs equimolarly (100µM per smMIP). smMIPs were produced by Integrated DNA technologies (IDT, Coralville, USA). Because the DNA ligase in the capture reaction needs a 5’ phosphorylated end, the smMIPs were first phosphorylated. The pool was phosphorylated in the following 35µL reaction composition: 29.53µL smMIP pool (100µM), 1.18µL T4 Polynucleotide Kinase (10U/µl) (New England Biolabs), 3.5µL 10X T4 DNA ligase buffer with 10mM ATP (New England Biolabs), and 0.79µL H_2_O. This reaction was incubated for 45 min at 37°C, followed by an inactivation step at 65°C for 20 min, and storage at -20°C until further use. The final concentration of each smMIP in this pool was 0.0286µM (100µM x 0.01µL / 35µL).

1. *Capture*

After MSRE digest, methylated CpG regions remained intact and were captured by the smMIPs through hybridization of the smMIP binding arms to the complementary genomic sequence. Elongation and ligation of the smMIP created a circular DNA fragment. In all capture reactions, some smMIPs were ligated without a 50nt insert. These so-called empty smMIPs were filtered out later.

For the capture reaction, a ratio of 800 smMIP copies per DNA target copy was chosen based on previously published research and optimization experiments. The volume of the phosphorylated smMIP pool to add was calculated as described by Arts *et al*. (19) and resulted in a volume of 0.000766µL. The phosphorylated pool was diluted using EB buffer (Qiagen, Hilden, Germany) for practical reasons.

To each 10µL digestion reaction, a 15µL capture mastermix was added (on ice), containing 7.66µL of the 1/10,000 smMIP pool dilution, 2.5µL 10X Ampligase DNA Ligase buffer (Lucigen), 0.032µL dNTPs (0.25nM) (Invitrogen), 0.32 Hemo Klentaq (10U/µL) (New England Biolabs), 0.01µL Ampligase DNA Ligase (100U/µL) (Lucigen), and 4.48µL H_2_O. This reaction was incubated for 10 min at 95°C and for 21.5 hours at 60°C. After incubation, the reaction was cooled down on ice for a few minutes and the exonuclease treatment was performed immediately.

1. *Exonuclease treatment*

Subsequently, an exonuclease treatment was performed. In this manner, all linear fragments such as unbound smMIPs or original DNA strands were degraded, and only circular fragments stayed intact. Removal of these linear fragments was important to specifically amplify the ligated smMIPs in the subsequent PCR reaction.

For this reaction, 2µL of EXO treatment mastermix was added to each capture reaction (on ice), containing 0.5µL EXO I (20U/µL) (New England Biolabs), 0.5µL EXO III (100U/µL) (New England Biolabs), 0.2µL 10X Ampligase DNA Ligase buffer (Lucigen), and 0.8µL H_2_O. Incubation was performed for 45 min at 37°C, followed by a heat inactivation step of 2 min at 95°C. The reaction was stored at 4°C until further use.

1. *qPCR and PCR amplification*

Before PCR amplification was initiated, a qPCR experiment was executed to determine the number of PCR cycles needed for a specific smMIP pool. For this one-time qPCR, 20µL of a mastermix containing 12.5µL iProof (Bio-Rad), 0.125µL forward primer (100µM), 0.125µL reverse primer (100µM), 0.125µL SYBR Green (Invitrogen) and 7.125µL H_2_O was added to 5µL of each exonuclease treated sample. The following PCR protocol was used: 30s at 98°C, 35 cycles of [10s at 98°C, 30s at 60°C, 30s at 72°C], and 2 min at 72°C.

Thereafter, the circular fragments were amplified by PCR using primers complementary to the universal PCR primer sites in the common smMIP backbone. The primers contained a unique barcode for each sample so many samples could be combined into a single library. The primers also contain a P5 or P7 oligo to allow binding to the Illumina flow cell (suppl figure 1).

The protocol was optimized by executing multiple PCRs reactions. Performing four replicate PCRs allows the effective use of all input DNA. As such, the number of PCR cycles was reduced, leading to less PCR duplicates and increasing the percentage of unique sequencing reads.

For the PCR, a mastermix was made per 192 samples. Each mastermix contained a forward primer identical per 192 samples. In total, 4 different forward primers were combined with 192 unique reverse primers. Firstly, a total of 18.75µL mastermix containing 12.5µL 2X iProof (Bio-Rad), 0.125µL forward Primer (100µM) and 6.125µL H_2_O was added to 6.5µL of the exonuclease treated samples. Secondly, 1.25µL of a unique reverse primer (10µM) was added to each sample. The PCR reaction was repeated four times, to use all exonuclease treated product. As such, less PCR cycles were needed to still obtain enough input DNA for sequencing, resulting in less PCR duplicates. The following PCR protocol was used: 30s at 98°C, 19 cycles of [10s at 98°C, 30s at 60°C, 30s at 72°C], and 2 min at 72°C. The products were stored at 4°C until further use.

Forward primer: AATGATACGGCGACCACCGAGATCTACACNNNNNNNNATACGAGATCCGTAATCGGGAAGCTGAAG

Reverse primer: CAAGCAGAAGACGGCATACGAGATNNNNNNNNACACGCACGATCCGACGGTAGTGT

1. *Pooling, bead purification and sequencing*

Finally, all samples were pooled and purified. The bead purification aims to remove superfluous enzymes, buffers, dNTPs and unnecessary fragments. Low amounts of DNA at the start of the protocol, result in increased amounts of empty smMIPs formed during the capture reaction. These empty smMIPs are formed by ligation without insert elongation. To make optimal use of the sequencing capacity, the empty smMIP fragments are removed before sequencing.

The total of 26.5µL PCR product from each sample was pooled per 24 samples. AMPure XP beads (Beckman Coulter) were added in 1:1 volume ratio, and the reaction was washed twice with 70% ethanol. Subsequently, the DNA of 96 samples was eluted in the same 100µL of TE buffer (0.01M Tris-HCl, 0.001M EDTA). A second purification step was executed to concentrate the pool and to lower the amount of smMIPs that did not capture any target (empty smMIPs), increasing sequencing capacity for correctly captured smMIPs. This is especially important for low DNA input amounts, as empty smMIPs are more prominent here. In the second purification step, 25µL of four 1x-purified pools with the same samples from four replicate PCRs were pooled, and beads were added in a 1:1 volume ratio. This reaction was washed twice with 70% ethanol and eluted in 40µL TE buffer. Optionally, more purification steps could be executed in case there is still an excess in amount of empty smMIPs. Finally, all individual pools were combined into the final NGS library.

Subsequently, concentrations and fragment lengths of the purified pools were determined by a High Sensitivity D1000 Kit on the TapeStation (Agilent). The concentrations of the region between 100bp and 1000bp were used. The final NGS library was sequenced on a High Output Kit on an Illumina NextSeq 550 system. 1.2pM of the NGS library was loaded and 5% of PhiX DNA was spiked into the library. Libraries were paired-end sequenced (2x75nt) using custom MIPs primers (suppl figure 1). For the validation experiment to evaluate the repeatability, the final library was sequenced using a Nano v2 kit on the Illumina MiSeq System.

**Supplementary methods: ddPCR assays**

IMPRESS was validated using ddPCR for 3 target sites. Two distinct assays were developed, where a triplex assay combined two target sites and a reference site and a duplex assay combined one target site with a reference site. The details of the assays are described below:

1. *Primer and probe design and ddPCR assay development*

Albumin was used as a reference target, for which the primers and the probe were already described by Boeckx *et al*. (1). For the probe, the FAM dye was changed to the SUN dye. Primers for the targets were designed by Primer3Plus version 0.4.0 using *in silico* bisulfite converted DNA through serial cloner version 2.6.1, or using MethPrimer. Non-fluorescent quencher, minor groove binder (NFQ-MGB) probes were manually designed. All primers and probes were ordered from IDT.

After optimization using temperature and concentration gradients, following parameters were found to be optimal for the two distinct assays:

| Target | Annealing Temp (°C) | Primer concentration (nM) | Probe concentration (nM) |
| --- | --- | --- | --- |
| Triplex - T1 | 55°C | 900 | 450 |
| Triplex - T2 |  | 900 | 680 |
| Duplex - T3 | 58°C | 900 | 1400 |
| Albumin | See target | 900 | 900 |

1. *ddPCR workflow*

Bisulphite converted DNA (EZ DNA methylation kit, Zymo Research) was used for DNA methylation detection. For the ddPCR, the recommended protocol and guidelines from BioRad were used.

Analyses after read-out of the droplets was done using the QuantaSoft™ Analysis Pro software version 1.0.596. Clusters for droplets were assigned manually. Cut-offs of 1) at least 10,000 accepted droplets and 2) at least 1,000 droplets for albumin were set for analyzing all ddPCR experiments. Samples that did not reach the cutoff values, were repeated.

1. *Calculations*

To determine the methylation level of a sample, normalized numbers of accepted droplets were used. The theoretical number of 20,000 droplets (max. number that can be obtained using the QX200™ system) was used for normalization. The limit of blank (LOB) was subtracted from the positive droplets to obtain the corrected methylation level. To summarize, the corrected methylation level was calculated as following:

$$Corrected methylation level=\frac{(\frac{\#positive droplets target-LOB}{\#accepted droplets target}*20 000)}{(\frac{\#positive droplets reference-LOB}{\#accepted droplets reference}*20 000)}*100$$

To determine sensitivity and specificity, receiver operator characteristic (ROC) curves were plotted and cross validated AUCs were calculated in R. These parameters were compared to the IMPRESS to validate the latter.

**Supplementary methods: Determination of the amount of input DNA**

1. *For digestion*

The standard protocol for an MSRE digest requires 1µg of input DNA in a final reaction volume of 50µL with 10 units of each enzyme. To test the possibility to lower the input amount, we tested different reaction conditions (suppl Figure 6a). The digested samples were amplified by qPCR with primer pairs hybridizing around the MSRE recognition sites. The results show that all tested reaction conditions performed similarly, as the Ct values ranged from 20.4 to 25.1 for primer pair 1, from 22.8 to 25.1 for primer pair 2 and from 28.0 to 30.8 for primer pair 3, while the undigested samples reached the threshold in the range of 11.0 to 12.7 cycles. The results also indicate the feasibility of lowering input DNA to 5ng.

1. *For capturing and sequencing*

To test the possibility of the smMIP capturing and sequencing with varying quantities of input DNA, the performance of a reaction starting from 10ng and 20ng was compared to the standard 100ng for smMIP-sequencing (15). gDNA from two blood samples and two cell lines was MSRE digested, captured by smMIPs and sequenced following our protocol. The sequencing results were analyzed using our in-house developed pipeline and read counts were obtained for the 2,331 CpG smMIPs and 600 reference smMIPs. To normalize the DNA input, the sum of CpG smMIP counts was divided by the sum of reference smMIP counts for each sample. This value is assumed to be higher in samples methylated for our targets (i.e. tumor samples) and lower in samples unmethylated for our targets (i.e. normal samples). Blood samples had an average of 0.18, 0.19 and 0.19 normalized counts for 100ng, 20ng and 10ng respectively, while cell lines had an average of 1.10, 1.23 and 1.26 counts respectively (suppl Figure 6b). This demonstrates that the results of our technique are consistent between an input amount of 10ng, 20ng and 100ng.

**Supplementary methods: Calculated percentage after spike-in**

To mimic the presence of circulating tumor DNA (ctDNA) in cfDNA, DNA from three tumor cell lines was sheared into fragments of 150-500bp using the Covaris. They were spiked into cfDNA samples in different percentages (different conditions): 0% (only cfDNA), 20%, 40%, 60%, 80% and 100% (only sheared cell line DNA). A total of 5ng DNA was used for each condition.

These mock ctDNA-cfDNA mixes were used for the IMPRESS protocol. After data quality control and mapping, read counts were obtained for CpG smMIPs and reference smMIPs in each sample. Normalized counts were calculated by dividing the sum of CpG smMIPs by the sum of reference smMIPs in each sample. For each cell line, a linear regression was plotted through the mock ctDNA-cfDNA samples. The following linear regression equations were obtained, with x being the theoretical percentage spike-in and y being the normalized count:

| HCT116 | y = -0.6297x + 4.3038 |
| --- | --- |
| SKHEP1 | y = -0.44x + 2.9083 |
| Cal27 | y = -0.5934 + 3.6367 |

These functions were used to predict the normalized counts value (y) for the 100% cell line samples (x = 100) corresponding to the linear regression line. This was necessary because in this way, not only the 100% cell line sample was determinative for the normalized counts value, but all the samples used for the linear regression line were.

Subsequently, for each sample, the normalized count was divided by the calculated 100% normalized counts value obtained from the linear regression from the corresponding cell line, to calculate the ratio (calculated percentage). This was done because the normalized counts differed per cell line and by calculation of the ratio, comparison of the cell lines was made possible.

Lastly, the Pearson’s correlation coefficient of the calculated percentage (i.e. observed spike-in percentage) and the expected percentage (i.e. theoretical spike-in percentage) was calculated.

| **Cell line** | **HCT116** | | | | | | **SKHEP1** | | | | | | **Cal27** | | | | | |
| --- | --- | --- | --- | --- | --- | --- | --- | --- | --- | --- | --- | --- | --- | --- | --- | --- | --- | --- |
| Percentage spike-in | 0 | 20 | 40 | 60 | 80 | 100 | 0 | 20 | 40 | 60 | 80 | 100 | 0 | 20 | 40 | 60 | 80 | 100 |
| Norm count | 0.22 | 1.12 | 2.19 | 2.74 | 2.89 | 3.45 | 0.33 | 0.61 | 1.12 | 1.48 | 1.95 | 2.58 | 0.28 | 0.65 | 1.13 | 1.62 | 2.36 | 3.31 |
| Norm count from linear regression |  |  |  |  |  | 3.67 |  |  |  |  |  | 2.46 |  |  |  |  |  | 3.04 |
| Calculated percentage | 0.06 | 0.30 | 0.60 | 0.75 | 0.79 | 0.94 | 0.13 | 0.25 | 0.45 | 0.60 | 0.79 | 1.05 | 0.09 | 0.21 | 0.37 | 0.53 | 0.77 | 1.09 |
| Correlation | 0.9405 | | | | | | 0.9885 | | | | | | 0.9695 | | | | | |

1. Boeckx N, Op de Beeck K, Beyens M, Deschoolmeester V, Hermans C, De Clercq P, et al. Mutation and Methylation Analysis of Circulating Tumor DNA Can Be Used for Follow-up of Metastatic Colorectal Cancer Patients. Clin Colorectal Cancer. Elsevier Inc.; 2018;17:e369–79.


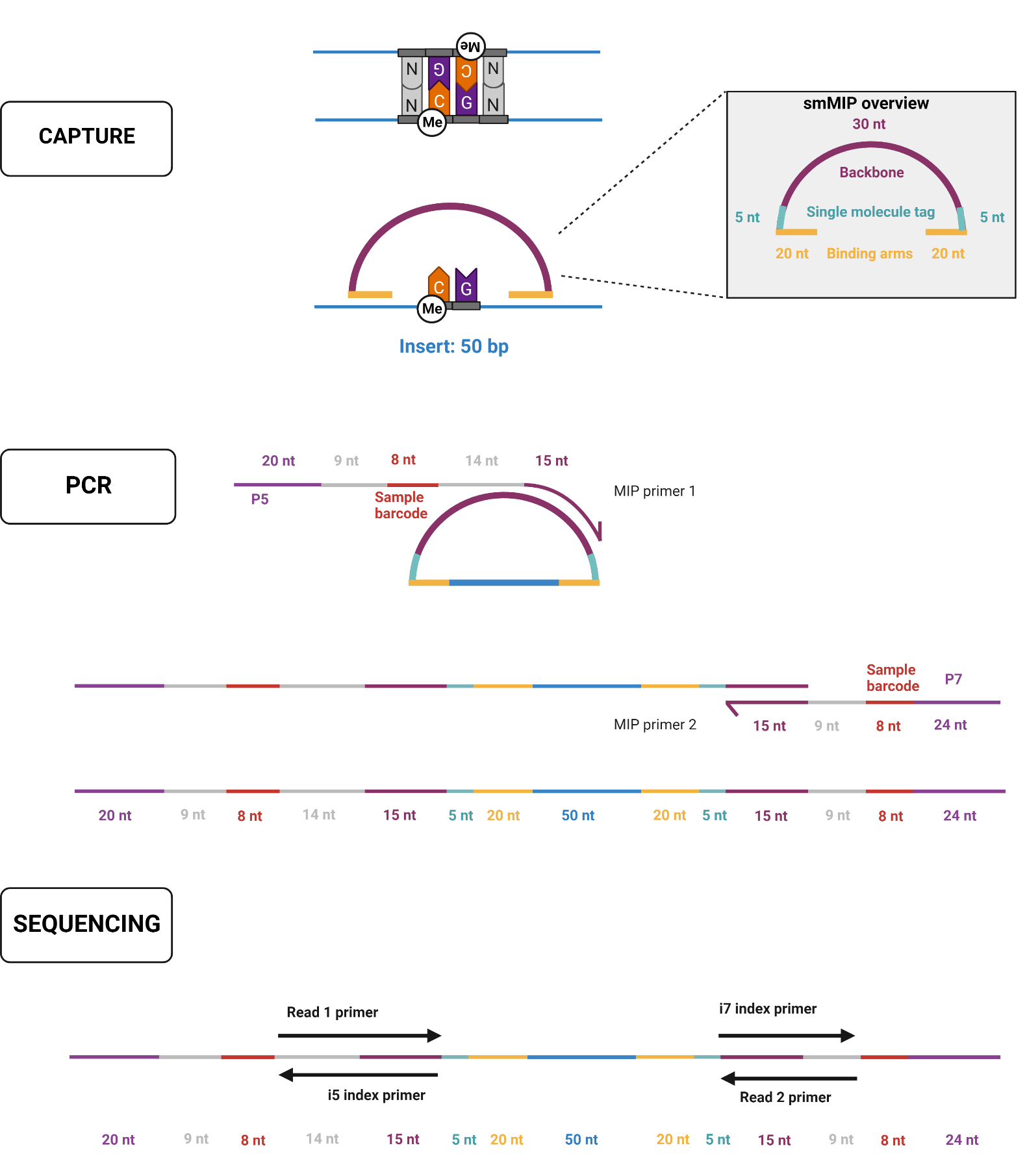


**Suppl. Figure 1 | Molecular overview of IMPRESS.** Numbers represent the nucleotide length of each fragment. Each smMIP contains a common smMIP backbone of 30nt (pink), single molecule tags of 5nt (green), and two binding arms of circa 20nt (yellow). During capture, the smMIP hybridizes to the target site, in order to have an insert length of 50nt. During PCR, MIP primers bind to the common smMIP backbone (pink). These primers additionally contain a sample barcode (red), a P5 or P7 oligo to bind the flowcell (purple) and supporting fragments (grey). During sequencing, the read 1 and 2 primers are used for the sequencing of the single molecule tag (green), the binding arm (yellow) and the insert fragment (blue), 75nt in each direction. The index primers are used for sequencing of the sample barcodes (red). Created with BioRender.com

**Suppl. Table 1 | Overview of the used cell lines.**

| **Name** | **Cancer type** |
| --- | --- |
| HT29 | Colorectal |
| HCT 116 | Colorectal |
| SK-HEP-1 | Liver |
| Miapaca | Pancreas |
| Cal27 | Head and neck |


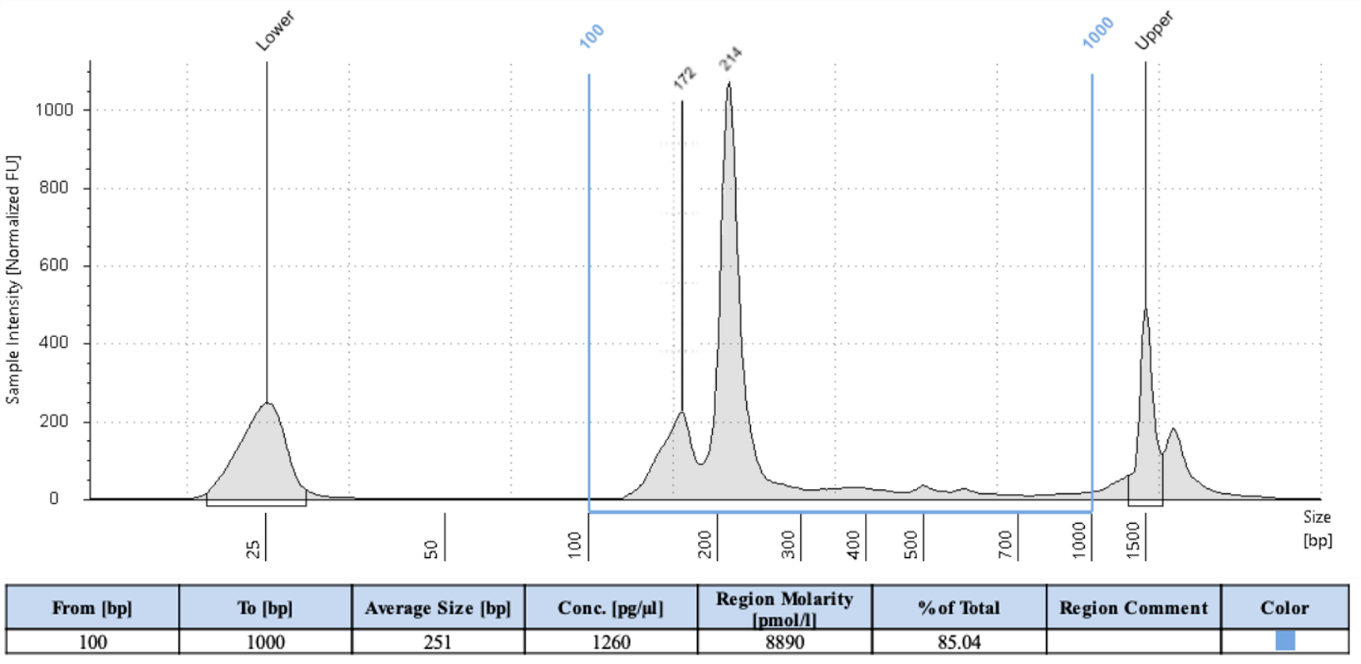


**Suppl. Figure 2 | Capillary electrophoresis analysis of the final NGS library using the Tapestation (Agilent).** The target peak is seen around 214bp (length of smMIP with insert = 222nt). 50bp to the left, the empty smMIPs are shown. For sequencing, the concentration of the pool is estimated based on the range from 100bp to 1000bp to avoid overclustering.

**Suppl. Table 2 | NGS quality parameters of NextSeq run.**

| **Read** | **Cycles** | **(Projected) Yield** **(Gbp)** | **Aligned PhiX (%)** | **Error Rate (%)** | **Intensity cycle 1** | **%> Q30**  **(Quality control)** | **Lane** | **Cluster PF (%)** | **Density** |
| --- | --- | --- | --- | --- | --- | --- | --- | --- | --- |
| **Read 1** | 75 | 35.20 | 3.40 | 0.19 | 7274.73 | 91.87 | **1** | 86.71 ± 0,67 | 212 ± 5 |
| **Read 2** | 8 | 3.33 | 0 | 0 | 6699.25 | 93.87 | **2** | 86.50 ± 0.63 | 211 ± 8 |
| **Read 3** | 8 | 3.33 | 0 | 0 | 4527.20 | 90.02 | **3** | 86.74 ± 0.61 | 211 ± 9 |
| **Read 4** | 75 | 35.20 | 3.33 | 0.28 | 6833.01 | 91.86 | **4** | 86.40 ± 0,77 | 210 ± 10 |
| **Non-index reads total** | 150 | 70.41 | 3.36 | 0.24 | 7053.87 | 91.86 |  |  |  |
| **Totals** | **166** | **77.07** | **3.36** | **0.24** | **6333.55** | **91.87** |  |  |  |

**Suppl. Table 3 | Filtered read counts per sample.**

See excel file


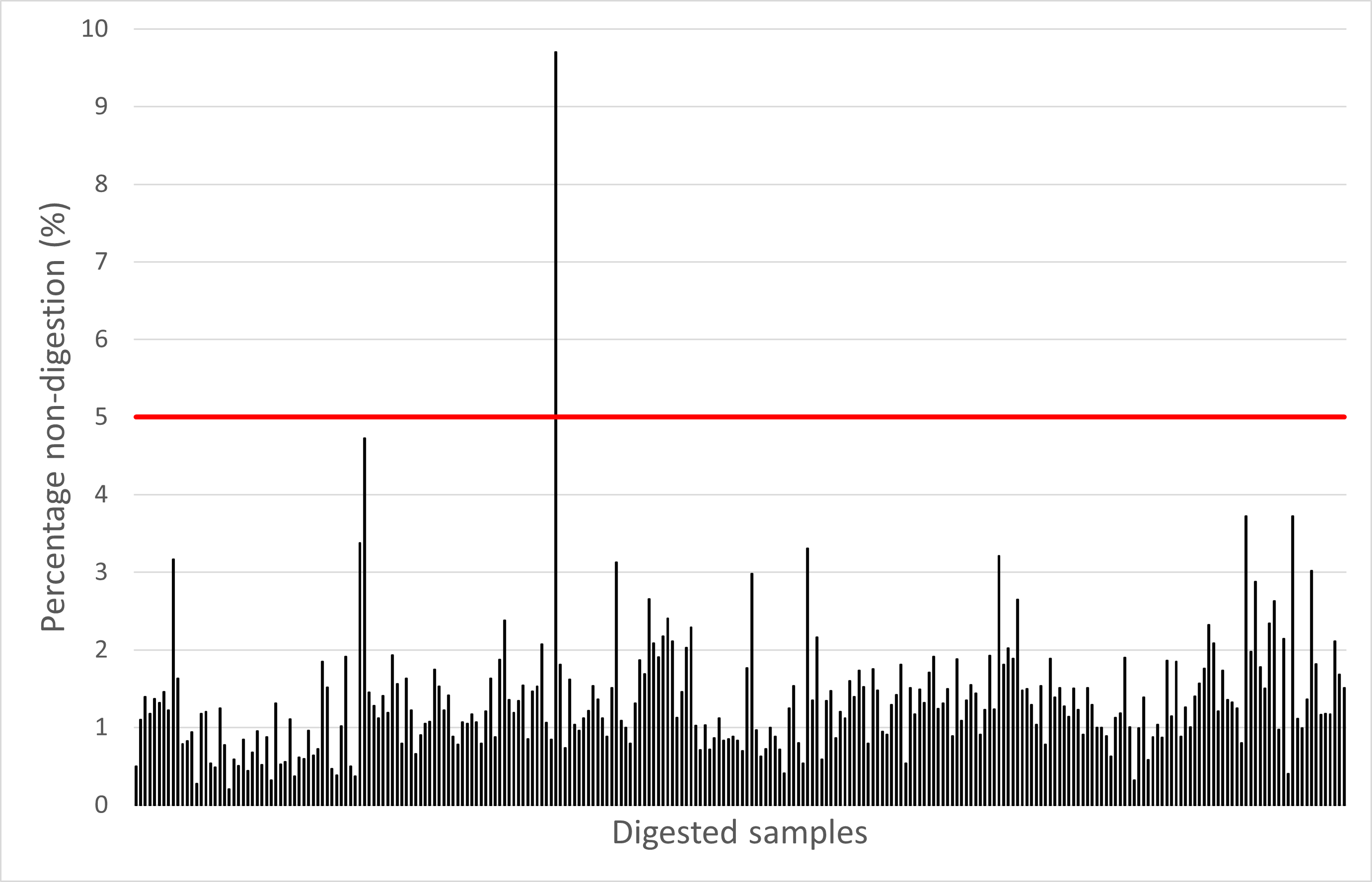


**Suppl. Figure 3 | Percentage of non-digestion in digested samples based on spiked-in lambda phage DNA.**


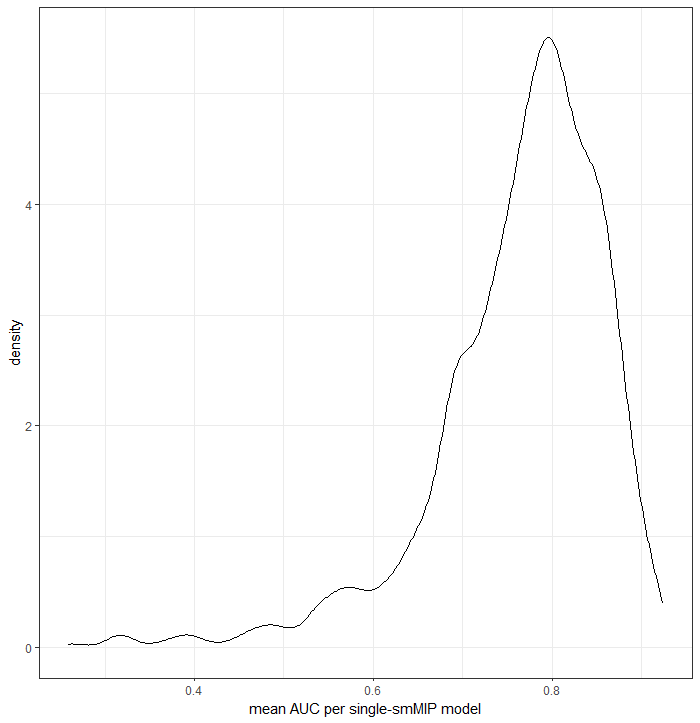


**Suppl. Figure 4 | Density plot of the mean cross validated AUC (cvAUC) values for each single smMIP model.** The distribution of cvAUC values is centered around 0.8 and is left-skewed. It is clear that the majority of smMIP models can make an accurate prediction.


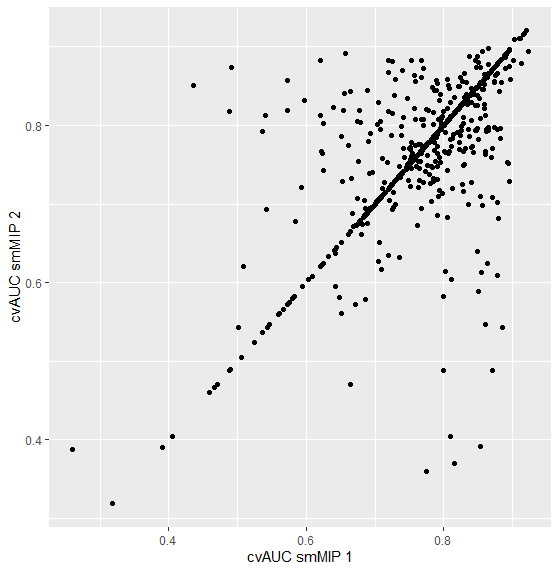


**Suppl. Figure 5 | Correlation of cvAUC values of all double-tiled smMIPs.** Selection of all CpG sites targeted by two smMIPs. For those CpG sites, cvAUC of one smMIP is in the X axis and the other in the Y axis. The pearson correlation coefficient is r = 0.668.

**Suppl. Table 4 | Genomic locations of 358 selected targets for final classifier model.** Locations are given in genome build 19.

See excel file

**Suppl. Table 4 | Sensitivities and specificities for the three selected target sites in both the IMPRESS assay and the ddPCR assay.** The sensitivities and specificities are calculated for each cancer type separately and for the overall analysis. Statistical analyses were conducted using the differences in proportions test and significant differences are indicated. ns = not significant, * = p-value ≤ 0.05, ** = p-value ≤ 0.01, *** = p-value ≤ 0.001 and **** = p-value ≤ 0.0001.

|  | **Target 1** | | | | | |  | **Target 2** | | | | | |
| --- | --- | --- | --- | --- | --- | --- | --- | --- | --- | --- | --- | --- | --- |
|  | **Sensitivity** | | | **Specificity** | | |  | **Sensitivity** | | | **Specificity** | | |
|  | ddPCR | IMPRESS |  | ddPCR | IMPRESS |  |  | ddPCR | IMPRESS |  | ddPCR | IMPRESS |  |
| Breast | 100% | 100% | ns | 100% | 100% | ns |  | 80.0% | 90.0% | ns | 100% | 100% | ns |
| Colorectal | 28.6% | 28.6% | ns | 100% | 100% | ns |  | 100% | 100% | ns | 50.0% | 60.0% | ns |
| Lung | 100% | 90.9% | ns | 100% | 100% | ns |  | 85.0% | 72.7% | ns | 100% | 100% | ns |
| Prostate | 100% | 93.3% | ns | 91.7% | 88.0% | ns |  | 85.7% | 86.7% | ns | 83.3% | 96.0% | ns |
| Pancreas | 82.4% | 85.0% | ns | 100% | 100% | ns |  | 76.5% | 80.0% | ns | 91.3% | 100% | ns |
| Liver | 75.0% | 69.2% | ns | 100% | 100% | ns |  | 75.0% | 61.5% | ns | 90.9% | 90.9% | ns |
| Esophagus | 100% | 100% | ns | 100% | 100% | ns |  | 90.0% | 90.0% | ns | 80% | 100% | ns |
| Head and neck | 53.8% | 61.5% | ns | 100% | 100% | ns |  | 69.2% | 69.2% | ns | 100% | 100% | ns |
| **Overall** | **83.5%** | **81.1%** | **ns** | **98.2%** | **97.4%** | **ns** |  | **81.6%** | **79.0%** | **ns** | **88.2%** | **94.7%** | **ns** |

|  | **Target 3** | | | | | |
| --- | --- | --- | --- | --- | --- | --- |
|  | **Sensitivity** | | | **Specificity** | | |
|  | ddPCR | IMPRESS |  | ddPCR | IMPRESS |  |
| Breast | 90.0% | 100% | ns | 100% | 100% | ns |
| Colorectal | 100% | 100% | ns | 50% | 0% | *** |
| Lung | 75.0% | 90.9% | ns | 100% | 95.5% | ns |
| Prostate | 35.7% | 73.3% | * | 91.7% | 92.0% | ns |
| Pancreas | 87.5% | 80.0% | ns | 95.7% | 91.7% | ns |
| Liver | 50% | 76.9% | ns | 100% | 90.9% | ns |
| Esophagus | 100% | 100% | ns | 80% | 80.0% | ns |
| Head and neck | 61.5% | 76.9% | ns | 100% | 75.0% | ns |
| **Overall** | **72.3%** | **87.2%** | ******* | **91.8%** | **83.3%** | **ns** |


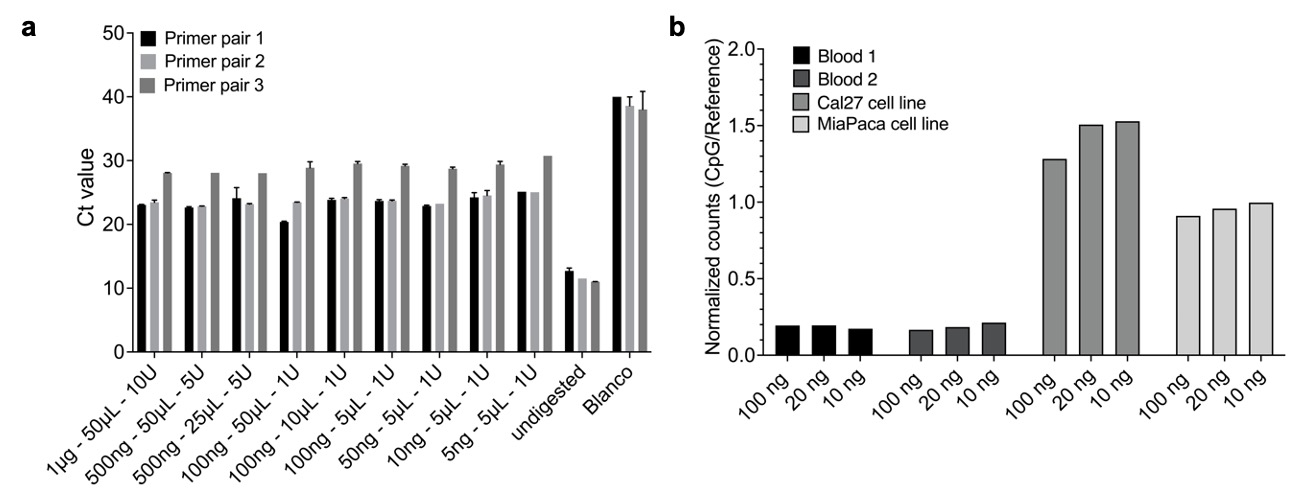


**Suppl. Figure 6 | Determination of the amount of input DNA. a) Results of the qPCR with lambda DNA digested in different reaction conditions**. Lambda DNA was digested in different sample conditions. These conditions are provided in the x-axis: the input amount of lambda DNA, the reaction volume of the digestion reaction, and the amount of enzyme units of each of the enzymes. The undigested samples were treated the same as the digested samples, except for the addition of the restriction enzymes. 1ng of all samples was amplified with four primer pairs, each hybridizing around one of the MSRE restriction sites. One primer pair failed and was excluded from the graph. The bars represent the average Ct value of two duplicate samples. **b) Normalized counts for different amounts of input DNA.** 100ng, 20ng and 10ng of genomic DNA from two blood samples and two cell lines (Cal27 and MiaPaca) was used as input for IMPRESS. Plotting was performed using GraphPad Prism.
